# A simple and safe technique for reconstruction of the acromioclavicular joint

**DOI:** 10.4103/0973-6042.68414

**Published:** 2010

**Authors:** Paul R. P. Rushton, James M. Gray, Tim Cresswell

**Affiliations:** Department of Orthopedic Surgery, Royal Derby Hospital, Uttoxeter Road, Derby, DE22 3NE, England

**Keywords:** Acromioclavicular, dislocation, reconstruction, trauma

## Abstract

Surgical reconstruction of the dislocated acromioclavicular joint often requires exposure and instrumentation of the coracoid. This carries risks to the surrounding neurovascular structures. We present a safe and simple technique of primary fixation of the acromioclavicular joint, relying on mechanical principles and biological repair, without the need for metalwork. By avoiding the coracoid we hope this approach will appeal to the general orthopedic surgeon. We have found that this technique is suited to both acute and chronic acromioclavicular joint dislocation.

## INTRODUCTION

**Figure 1:**
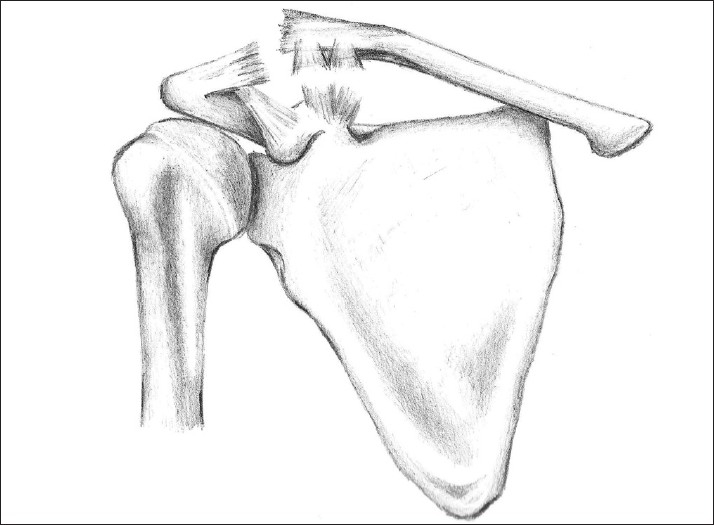
During anatomically complete dislocations of the AC joint, rupture of the acromioclavicular and coracoclavicular ligaments allows displacement of the clavicle

Acromioclavicular (AC) joint injuries are most common among male sportsmen under 30 years of age, and account for 3 – 12% of the shoulder injuries seen by orthopedic surgeons.[1,2] AC dislocations commonly follow a fall onto the point of the shoulder with the arm adducted. Most surgeons classify the injury using the Rockwood system, which is dependent on the damage to the AC joint capsule and ligaments, as well as the coracoclavicular ligaments.[2] Type I and II injuries are usually treated conservatively. Type IV – VI injuries usually necessitate surgery, to limit deformity, pain, and weakness. Debate over the treatment of type III injuries is longstanding, but recent research favors conservative management for most cases.[3] Anatomically complete dislocations (type III – VI) involve rupture of both the AC and coracoclavicular ligaments [Figure 1].

Since the first reported procedure for AC joint repair by Cooper in 1861,[4] numerous techniques have been used to treat AC dislocation. The techniques have tended to fall into five groups: primary fixation of the AC joint, secondary stabilization linking the distal clavicle and the coracoid, distal clavicle excision, dynamic stabilization or a combined approach. Despite such attention, the lack of a dominant procedure suggests that the ideal repair is yet to be found. Our technique involves primary fixation of the AC joint. Unlike other techniques the reconstruction avoids metalwork crossing the joint, instead relies on biological repair. A strategy recommended by previous research.[5]

## TECHNIQUE

This is a two-stage technique; first a tendon graft is harvested from the forearm. Regional anesthesia is our routine, so the tendon is usually harvested from the same side as the AC joint being reconstructed. The joint is subsequently repaired using the graft together with high strength non-absorbable suture material, such as Ultrabraid (Smith and Nephew), to provide a strong immediate fixation while biological healing occurs.

### Tendon harvest

The whole upper limb is prepared in the usual manner. The tendon of palmaris longus is harvested via conventional methods and placed in saline. In those lacking palmaris longus we use half the tendon of flexor carpi radialis. The wounds can then be closed and dressed.

### Joint reconstruction

**Figure 2:**
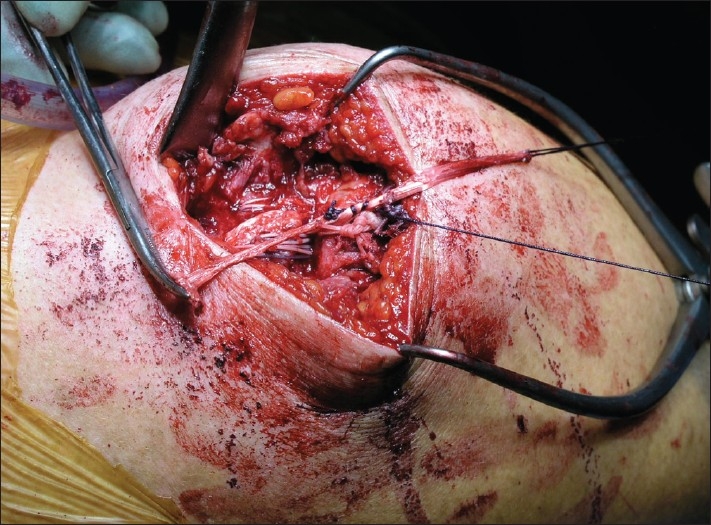
Photograph depicting tendon graft being drawn into the reconstruction

**Figure 3:**
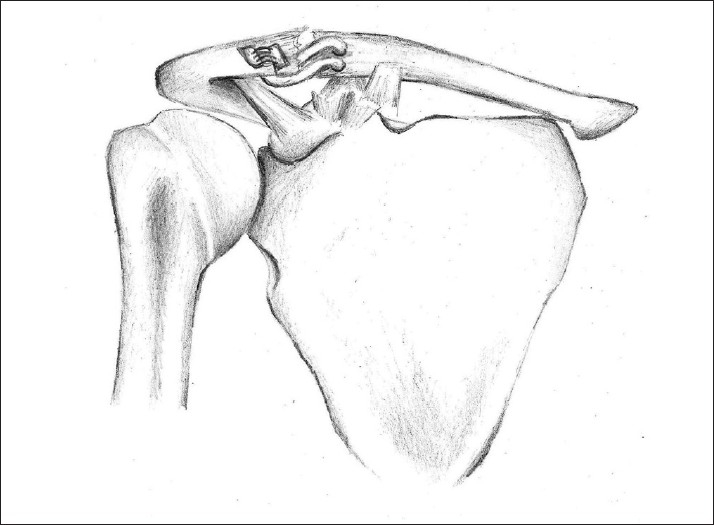
Diagram of completed reconstruction. Tendon graft and high tensile suture span the AC joint. There is no attempt to formally reconstruct the coracoclavicular ligaments

**Figure 4:**
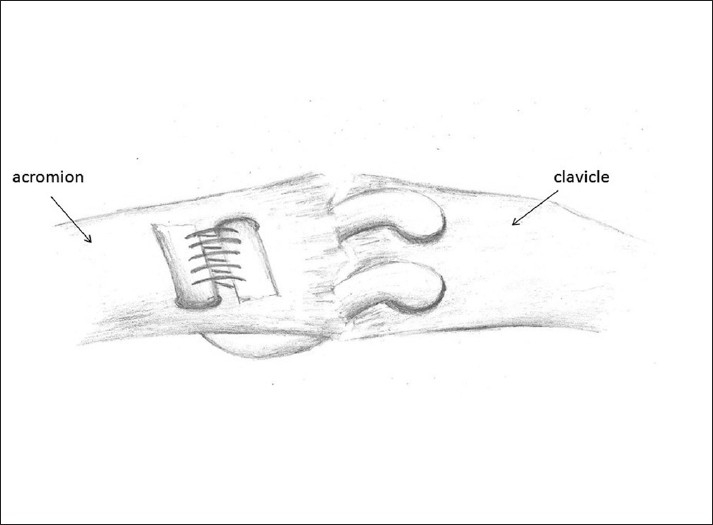
Superior view of repair. Tendon and sutures emerge on the superior aspect of the clavicle and traverse the AC joint, before emerging on the superior aspect of the acromion, where they are tied

**Figure 5:**
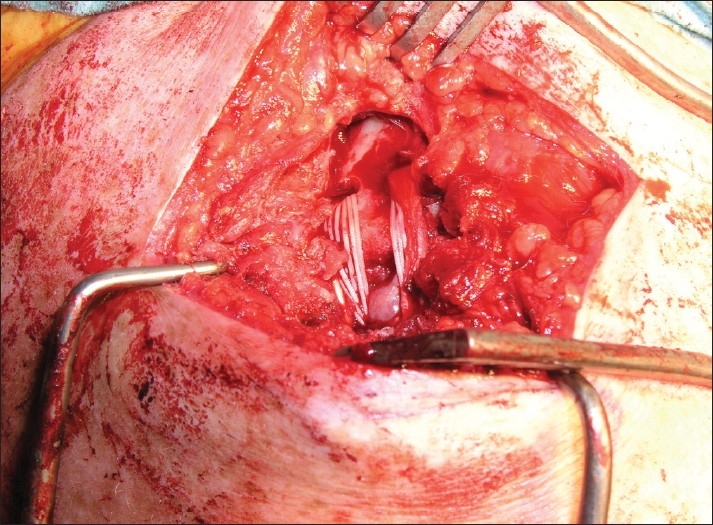
Photograph of completed reconstruction

A transverse incision is made over the AC joint. The capsule is opened longitudinally and the dislocation inspected. An excision of the distal clavicle of approximately 7 mm is performed and the acromion roughened up. Two 4 mm drill holes are made in the clavicle and acromion, about 1 cm away from the AC joint. Loop PDS (Ethicon) is passed down through the superior aspect of the posterior hole in the acromion and retrieved in the AC joint. The loop PDS is then passed down through the superior aspect of the posterior hole in the clavicle and retrieved underneath the clavicle. It is then passed up through the inferior aspect of the anterior hole in the clavicle and brought out to the superior surface of the clavicle. Finally, the PDS is passed down through the AC joint, and through the inferior aspect of the anterior hole in the acromion and brought out of the superior aspect of this hole. This loop of PDS connects all four drill holes. The PDS is used as a shuttle for four strands of Ultrabraid (doubled back of themselves) and the palmaris graft. Overall eight strands of Ultrabraid and the tendon graft, link each drill hole. The sutures are tied and the graft sutured to itself on the superior aspect of the acromion [Figures 2–5]. Additional ties of Ultrabraid can be performed to increase tension on the joint as necessary.

The capsule is closed with absorbable sutures, the fat and skin closed in the standard fashion and the arm placed in a poly sling. The patient can be discharged after the immediate postoperative period and is instructed to avoid lifting and pendular exercise for six weeks. After this period the patient is reviewed in the clinic.

## DISCUSSION

We have used this technique for both acute and chronic type III – V dislocations. Patient outcomes have been favorable. A full outcome analysis is ongoing.

Many techniques used to repair AC dislocations necessitate exposure and often instrumentation of the coracoid, such as, transfer of the coracoacromial ligament in a Weaver and Dunn[6] based approach or to anchor some sort of coracoclavicular fixation such as a Bosworth screw[7] or suture anchor.[8] Open procedures involving the coracoid require a larger incision and greater soft tissue dissection than AC joint surgery alone. Such invasive procedures are becoming harder to justify with the increasing use of arthroscopic approaches. Even with the large exposure, the visualization of the coracoid, especially medially, can be limited. Hence, these approaches carry a greater risk of damage to nearby neurovascular structures.[1] Thus, we present a technique for primary reconstruction of the AC joint, which avoids the exposure and risk of procedures involving the coracoid.

This reconstruction has been designed with mechanical principles in mind. The repair provides an upward force on the acromion, which tends to sag downward with the weight of the arm. A downward force is applied to the distal clavicle, which usually rises superiorly under the force of the trapezius muscle. Furthermore, the repair follows the engineering principle of a four bar-gate, known for its stability.

Given that our technique does not repair coracoclavicular ligaments, it cannot be considered a true anatomic reconstruction. Our technique only reconstructs the AC ligaments, which have been shown to resist all movements of the AC joint at what can be considered physiological loading and displacement, as well as being the primary stabilizer in the anterior–posterior rotation of the clavicle on the scapula.[9] Reconstructions involving high tensile sutures like Ultrabraid have been shown to behave similarly to native ligaments biomechanically.[5] Given this, together with biological healing from the graft, we hope to create a biomechanically sound reconstruction. In not repairing the coracoclavicular ligaments, we acknowledge the loss of some stability, particularly during superior displacement of the clavicle and against compressive forces.[9] However, we feel that the simplicity and safety gained by avoiding the coracoid outweighs this increased stability.

AC surgery has historically been found to have certain issues, which we aim to avoid. Techniques using metalwork across the AC joint such as hook plates or screws from clavicle to coracoid necessitate a second procedure to remove this metalwork, to avoid the dangers of metalwork migration away from the joint.[2] This lengthens the overall hospital stay and theater time, increasing risk and costs. Fibers such as Ultrabraid do not seem to suffer from this migration and have been used successfully in AC joint reconstructions.[10] AC surgery has also been found to suffer higher rates of post-traumatic arthritis. By avoiding penetrating the articular cartilage and using a small distal clavicle excision, we hope this will be limited. The excision will also provide a bleeding surface, aiding biological repair.

In summary, we have successfully used a novel technique for reconstruction of the dislocated AC joint. This technique of primary acromioclavicular fixation avoids the associated risks of coracoid exposure and instrumentation. The reconstruction relies upon biological repair and biomechanical principles to achieve a stable AC joint.
